# Integrated analysis and experiments uncover the function of disulfidptosis in predicting immunotherapy effectiveness and delineating immune landscapes in uterine corpus endometrial carcinoma

**DOI:** 10.3389/fimmu.2024.1454730

**Published:** 2024-10-09

**Authors:** Lei Han, Yilin Li, Yanjie Yu, Guo Liu, Xiangqian Gao, Fei Wang, Weiwei Chen, Huishu Xu, Baolin Zhang, Yingjiang Xu, Yitong Pan, Yu Huang, Ping Yi

**Affiliations:** ^1^ Department of Obstetrics and Gynecology, The Third Affiliated Hospital of Chongqing Medical University, Chongqing, China; ^2^ Department of Reproductive Medicine, Binzhou Medical University Hospital, Binzhou Medical University, Binzhou, Shandong, China; ^3^ Department of Obstetrics and Gynecology, Sichuan Provincial People's Hospital, School of Medicine, University of Electronic Science and Technology of China, Chengdu, Sichuan, China; ^4^ Department of Ultrasound, Binzhou Medical University Hospital, Binzhou Medical University, Binzhou, Shandong, China; ^5^ Department of Obstetrics and Gynecology, Binzhou Medical University Hospital, Binzhou Medical University, Binzhou, Shandong, China; ^6^ Department of Pathology, Binzhou Medical University Hospital, Binzhou Medical University, Binzhou, Shandong, China; ^7^ Medical Research Center, Binzhou Medical University Hospital, Binzhou Medical University, Binzhou, Shandong, China; ^8^ Department of Interventional Vascular Surgery, Binzhou Medical University Hospital, Binzhou Medical University, Binzhou, Shandong, China; ^9^ Key Laboratory of Genomic and Precision Medicine, Beijing Institute of Genomics, Chinese Academy of Sciences, Beijing, China

**Keywords:** disulfidptosis, uterine corpus endometrial carcinoma, tumor microenvironment, LRPPRC, immunotherapy

## Abstract

**Introduction:**

Recently, a novel type of metabolic-regulated cell demise titled disulfidptosis has been discovered. Studies have demonstrated its importance in immune responses against cancer and its impact on the proliferation of cancer cells. Nonetheless, the precise mechanism and roles of disulfidptosis are not fully understood, particularly regarding the prognosis for individuals with uterine corpus endometrial carcinoma (UCEC).

**Methods:**

In this research, a distinctive disulfidptosis pattern was developed in UCEC, and by utilizing Non-negative Matrix Factorization (NMF) on 23 disulfidptosis related genes within the TCGA database, 3 distinct subgroups were distinguished. To collect data, we acquired gene expression profiles, somatic mutation information, copy number variation data, and corresponding clinical data from the TCGA and GEO database, specifically from UCEC patients. Cell line experiments and immunohistochemical (IHC) staining were conducted to validate the role of the LRPPRC in proliferation, migration and invasion.

**Results:**

The genetic features and immune microenvironment of these subgroups were examined. It is worth mentioning that these subgroups offer important insights into comprehending the tumor microenvironment (TME) and the response of patients to immunotherapy and chemotherapy. Moreover, a disulfidptosis model was developed and validated, demonstrating a high level of accuracy in predicting the prognosis and outcomes of immunotherapy in UCEC patients. Additionally, a novel biomarker, LRPPRC, was identified, which can server as a promising predictor for forecasting prognosis in UCEC patients, with validation through tissue microarray staining and cell line experiments.

**Discussion:**

This study has designed a classification system and a disulfidptosis model for UCEC, in addition to identifying a new biomarker, LRPPRC, for UCEC. These advancements serve as reliable and positive indicators for predicting outcomes and the efficacy of immunotherapy for each UCEC patient.

## Introduction

Uterine Corpus Endometrial Carcinoma, recognized globally as one of the most frequent gynecological malignancies, represents 3.8% of all cancer cases in women and 27.5% of gynecological cancers (1). Early-stage UCEC is notable for often lacking obvious clinical symptoms, leading to its frequent diagnosis at advanced stages marked by aggressive disease progression and resulting in unfavorable outcomes (2). Despite advancements in therapy, the 5-year survival rate for those treated remains between 70% and 80% (3). Therefore, the challenge at this stage lies in identifying novel diagnostic methodologies and prognostic evaluation strategies that can aid in personalized therapeutic interventions.

Up to now, the ongoing progress in immuno-oncology and the development of immunotherapeutic treatments, such as immune checkpoint inhibitors (ICIs) and chimeric antigen receptor (CAR) T cell therapy, provide hopeful strategies to fight against cancer by triggering the body’s own immune response (4). Various modulators maintain the effectiveness of immunotherapy within a complex network. The tumor microenvironment represents a key factor impacting the success of immunotherapy, with the quantity and types of immune cells infiltrating the tumor and the makeup of these immune cells playing a crucial role in promoting antitumor immune responses (5, 6). Likewise, the levels of different cytokines, the presence of immune checkpoints, and the antigen presentation facilitated by MHC molecules are integral to the immune response elicited by immunotherapy (4). Nonetheless, the TME can vary considerably across different types of cancers and between individuals, possibly due to the diverse genetic makeup of tumors (7). Furthermore, the metabolic changes occurring within the TME influence antitumor immune responses, underscoring the importance of metabolic programs in shaping the TME (8).

Regulatory cell death (RCD) plays a crucial role in preventing tumorigenesis and progression as well as impacting the tumor microenvironment and immunotherapy (9). Disulfidptosis, the most recently discovered form of RCD, is distinct from other types as it is induced by glucose deprivation leading to the accumulation of small disulfide molecules (10). Mechanistically, cells with high levels of SLC7A11 expression require significant NADPH from the glucose-pentose phosphate pathway (GPP) to convert cystine to cysteine. Insufficient glucose supply results in a lack of REDOX power, causing an abnormal buildup of cystine or disulfide molecules and ultimately cell death, defining disulfidptosis as a metabolism-regulated form of cell death. Previous research has highlighted the prognostic significance of disulfidptosis in advanced cancers (11, 12). However, the impact of disulfidptosis on TME and immunotherapy outcomes in uterine corpus endometrial carcinoma is not well understood to date.

In this study, we constructed an innovative classification system utilizing genes related to disulfidptosis. We categorized all UCEC patients into three distinct clusters based on varying TME and genomic characteristics. Interestingly, these subclusters showed varying responses to both immunotherapy and chemotherapy. Moreover, we created and validated a disulfidptosis model designed to forecast the prognosis and immunotherapy outcomes for UCEC patients. Notably, our immunohistochemical staining using tissue microarrays, along with cell line experiments, revealed a correlation between the expression of LRPPRC and poor survival, as well as the invasion and proliferation of UCEC cells. This finding indicates that LRPPRC could serve as a novel potential target for immunotherapy in the future. In summary, our extensive analysis offers valuable insights into the role of disulfidptosis in the prognosis and immunotherapy of ovarian cancer.

## Materials and methods

### Data acquisition and evaluation

Initially, we acquired UCEC transcriptome datasets from the TCGA data portal, containing clinical variables like age, gender, stage, survival rates, and progression-free survival. We also obtained UCEC genomics data from the TCGA database, including somatic mutation and copy number variation details. Samples lacking survival data were excluded from the analysis. To explore the UCEC microenvironment, we retrieved five single-cell UCEC samples from the GEO database (accession: GSE173682) (13). For classification and modeling, we identified 23 disulfidptosis-related genes from the literature (10). Chemotherapy response data was retrieved using the R package TCGAbiolinks (v2.16.4). Additionally, we collected leukocyte and tumor-infiltrating lymphocyte fraction numbers calculated from H&E images of 13 TCGA tumor types by Saltz et al. (14).

### Prediction of immunotherapy response of UCEC

To determine the effectiveness of PD-1/CTLA4 immunotherapy, we began by calculating scores for tumor immune dysfunction and exclusion (TIDE) using the modified expression data from UCEC patients. We then analyzed the resulting expression profiles matrix via the TIDE database website to assess patient responses. Following this, we applied the submap algorithm available on the GenePattern website to compare the response probabilities across various groups.

### Gene set variation analysis and functional enrichment analysis

To investigate the variations in hallmark and immune-related signatures in UCEC patients, an analysis of pathway enrichment was conducted with the ‘fgsea’ R package. The gene sets were obtained from the MSigDB database (http://software.broadinstitute.org/gsea/msigdb/index.jsp). Additionally, an evaluation of the functional variances between patients in the high-DFDS group and those in the low-DFDS group was performed through Gene Ontology (GO) and Kyoto Encyclopedia of Genes and Genomes (KEGG) pathway enrichment analyses using the ‘gseGO’ and ‘gseKEGG’ R packages, respectively.

### Deciphering the immune landscapes

To ascertain the proportion of immune cells in each UCEC samples, the xCELL algorithm was utilized. The immune cell infiltration score in patients with UCEC was sourced from the TIMER2.0 database (15). For the evaluation of classical immune signatures and immune function scores, we opted for the single-sample Gene Set Enrichment Analysis (ssGSEA) algorithm available in the R package “GSVA.” This approach allowed us to measure the enrichment levels of gene sets related to immune functions for each sample quantitatively.

Subsequently, we utilized the R package ESTIMATE (v1.0.13) was employed to calculate the following parameters for all each sample within the TCGA-UCEC dataset: Stromal Score, Immune Score, and Tumor Purity.

### Development of a disulfidptosis model through machine learning analysis

To achieve the goal of checking for plagiarism, the given text has been revised as follows: To lower the complexity of the univariate Cox regression findings, Lasso regression was utilized. Redundancy was removed via 500 rounds of Lasso regression. Genes that appeared 500 times in these rounds were chosen for model development with the R software package glmnet. The next step involved constructing a model using multivariate Cox regression. Incorporating genes into the model involved stratifying them. The AUC for all possible gene combinations was calculated, and the model with the highest AUC was chosen as the final one. In the end, 11 genes related to disulfidptosis were identified as inputs for the final model. The DFDS score for each sample was then computed using the following formula:


$$DFDS_{\text{i}}={\text{α}}_{\text{j}}*\sum_{\text{j}=1}^{n}\exp_{\text{ji}}+\beta$$

In the equation, “exp” stands for the level of expression of the corresponding gene. The symbol “α” indicates the regression coefficient (coef) of the gene in the Lasso regression results, and “β” represents the adjustment coefficient. The DFDS (disulfidptosis score) is calculated by multiplying the expression level of every significantly associated gene in a specific sample by its corresponding regression coefficient, and then adding them together. The variable “i” denotes the sample, while “j” signifies the gene.

### Validation the performance of model

Patients were classified according to the ideal boundary set by the ‘survminer’ R program. Following this, we created the Kaplan-Meier plot and ROC graph utilizing the overall survival information and calculated the significance level. Any significance level below 0.05 was viewed as evidence of a substantial contrast between the elevated and reduced DFDS categories. The model forecasts originated from the DFDS, with AUC figures above 0.7 indicating a praiseworthy model performance.

### Evaluation of pathway activity using PROGENy

To assess the function of various pathways in patients with UCEC, we employed the PROGENy method (16), which is capable of inferring multiple pathway activities based on gene expression. This method entails using key genes that respond to pathways, extracted from a wide range of publicly accessible perturbation experiments.

### Cell culture and transfections

HEC-1-A cells were obtained from Wuhan Pricella Biotechnology Co., Ltd. They were cultured in HEC-1-A medium (CM-0099, Procell, Wuhan, China) and passaged every 2 days. All cells were maintained at 37°C with 5% CO2. Cells were plated at a density of 2×10^5 per well in 6-well plates and transfected with either 10 µl of overexpression plasmid (LRPPRC, GenePharma, China) or 10 µl of siRNA (LRPPRC, GenePharma, China). Lipofectamine 3000 was used to facilitate the transfection according to the manufacturer’s instructions.

### Proliferative assay

Cells were plated in a 96-well plate at a density of 3000 cells per well. After incubation for 0, 1, 2, 3, and 4 days, cell proliferation was assessed using the MCE Cell Counting Kit-8 (HY-K0301, CCK-8; MedChemExpress) following the manufacturer’s instructions. Absorbance was measured using a BioTek Synergy H1 hybrid microplate reader at wavelengths of 450 nm and 630 nm. Relative proliferation was calculated as (OD450 - OD630) Sample/(OD450 - OD630) Control.

### Colony formation assay

Cells were plated in 6-well plates at a density of 500 cells per well and cultured in HEC-1-A medium for 14 days, with the medium being changed every three days. After this period, cells were fixed with 4% paraformaldehyde (A500684, Sangon Biotech) and stained with crystal violet (A100528, Sangon Biotech) for 2 hours. Optical imaging was performed using an Olympus IX53 inverted fluorescence microscope equipped with an Olympus DP73 color camera (Olympus, Tokyo, Japan). Densitometry of each visual field was analyzed using ImageJ.

### Transwell assay

1×10^4^ cells were suspended in 200 μL of DMEM medium without FBS and seeded on the top chamber of transwell inserts (3422, Corning). The lower chambers contained HEC-1-A medium with 10% FBS. After incubation for 48 hours, the chamber was rinsed one times with PBS, then stained with crystal violet for 2 hours and rinsed again with PBS three times to obtain a transparent background. Plates were imaged using the IX53 inverted fluorescence microscope and the DP73 color camera.

### Wound healing assay

Different groups of HEC-1-A cells are seeded in 6-well plates at a density of 4×10^5^ per well prior to processing. The next day, scrape the cells with a 200 μl needle tip and incubate for 72 h with serum-free medium. Micrographs were taken before and after treatment, and the relative distance was calculated by subtracting the wound width after treatment from the width of the wound before treatment and quantified with ImageJ software.

### Quantitative real-time polymerase chain reaction

RNA was extracted using Trizol (TransGen Biotech, China) following the manufacturer’s instructions. The purified RNA was reverse transcribed into cDNA using the RevertAid First Strand cDNA Synthesis Kit (Fermentas, USA) according to the manufacturer’s guidelines. Polymerase chain reactions were conducted with 2× Taq Master Mix (Vazyme, China). The mRNA primers were as follows:

LRPPRC Forward: 5′-GGACGGCAAGAATGTGACCT-3′

Reverse: 5′-GGTCGTGCTCCAATTATAGCCT-3′

GAPDH Forward: 5-GTCACCAGGGCTGCTTTTAACTC-3′

Reverse: 5′-CAGCATCGCCCCACTTGATTTTG-3′

The relative expression level of the mRNA was expressed as 2-(△△CT) and normalized to GAPDH.

### Protein extraction and Western blot

Cells were collected and lysed with RIPA lysis buffer (Solarbio, R0020, Beijing, China) containing phenylmethanesulfonyl fluoride (Solarbio, P0100, Beijing, China) on ice for 30 minutes. After centrifugation at 12,000 rpm for 15 minutes, the supernatant was collected, and protein concentration was measured using a BCA Kit (Solarbio, PC0020, Beijing, China) according to the manufacturer’s instructions. Proteins were separated via sodium dodecyl-sulfate polyacrylamide gel electrophoresis (SDS-PAGE) (BOSTER, AR0047, Wuhan, China) and transferred to a polyvinylidene difluoride (PVDF) membrane (ISEQ00010, Millipore, Burlington, MA, USA). Membranes were incubated with primary antibodies (1:1000 dilution) overnight at 4°C, followed by incubation with secondary antibodies (1:2000 dilution) for 2 hours at 37°C. Enhanced chemiluminescence signals (ECL) (UElandy, S6009M, Suzhou, China) were detected using Image Lab software. An antibody against tubulin (BM1452, BOSTER) was used as an internal control. Primary antibodies specific to LRPPRC (A03264, BOSTER) were purchased from ABclonal. Secondary antibodies, HRP-linked goat anti-rabbit IgG (H+L; 511203) were obtained from ZENBIO, and goat anti-mouse IgG (H+L; BA1050) were obtained from BOSTER.

### Immunohistochemical staining

Endometrial cancer tissues collected during surgery were rinsed with saline and blood removed, then fixed in 4% paraformaldehyde solution for 24 h. The tissue blocks were placed in an embedding cassette and rinsed with running water for 30 min to wash away the fixative. Tissue was dehydrated using a tissue dehydrator (Leica HistoCore PEARL), xylene was used to remove the dehydrating agent and then the tissue was encased in hot paraffin wax and cooled on an ice bench for 4-6 h. The tissue block was then fixed on a slicer to be sectioned. Sections were floated in warm water, carried by slides and then placed in an oven at a constant temperature of 60°C for 6 hours, dried and set aside. Sections were deparaffinized by xylene 2 times, soaked sequentially in a graded ethanol solution, and then immersed in PBS and rinsed 2 times. The sections were then submerged in sodium citrate antigen repair solution to maintain the water bath temperature between 85°C and 95°C for 15 min, while naturally cooled to room temperature, rinsed in PBS for 2 times, sealed with endogenous peroxidase in 3% hydrogen peroxide methanol solution, rinsed in PBS for 2 times, and the membranes were broken by immersion in 0.1% Triton X100 solution for 8 min followed by 2 times of immersion in PBS. The membrane was broken by immersion in 0.1% Triton X100 solution for 8 min, followed by immersion in PBS for 2 times, and then goat serum was added dropwise after drying the PBS around the sample with absorbent paper for 40 min. After wiping off the excess PBS around the sample, the secondary antibody was added immediately and incubated at room temperature for 2 h. Then PBS was immersed for 5 times, and the excess PBS around the sample was wiped off for DAB color development, and then immersed in water to terminate the reaction. After hematoxylin staining and hydrochloric acid-ethanol differentiation, respectively, the samples were rinsed with water, dehydrated with gradient ethanol, and treated with xylene, then the samples were sealed with droplets of neutral dendrimer and observed with a light microscope.

### Statistical analysis

We utilized wilcoxon test to compare different attributes between high and low DFDS groups. Unsupervised clustering was performed on the gene expression matrix associated with disulfidptosis using the R package “NMF”. The somatic mutation landscape was assessed and displayed by employing the R package maftools (v1.0-2). Examination of diversity in clinical characteristics and response to immunotherapy among various groups was conducted using the Chisq test. Kaplan-Meier survival analysis was carried out to explore differences in survival between different subgroups, high- or low DFDS groups, with the significance of observed differences determined by the log-rank test. The strength of intercellular interaction between immune cells and malignant tumor cells was computed using cellphoneDB (17). Statistical significance was defined at a significance level of P or adjP< 0.05.

## Results

### Disulfidptosis-associated genotyping of uterine corpus endometrial carcinoma

**Figure 1:**
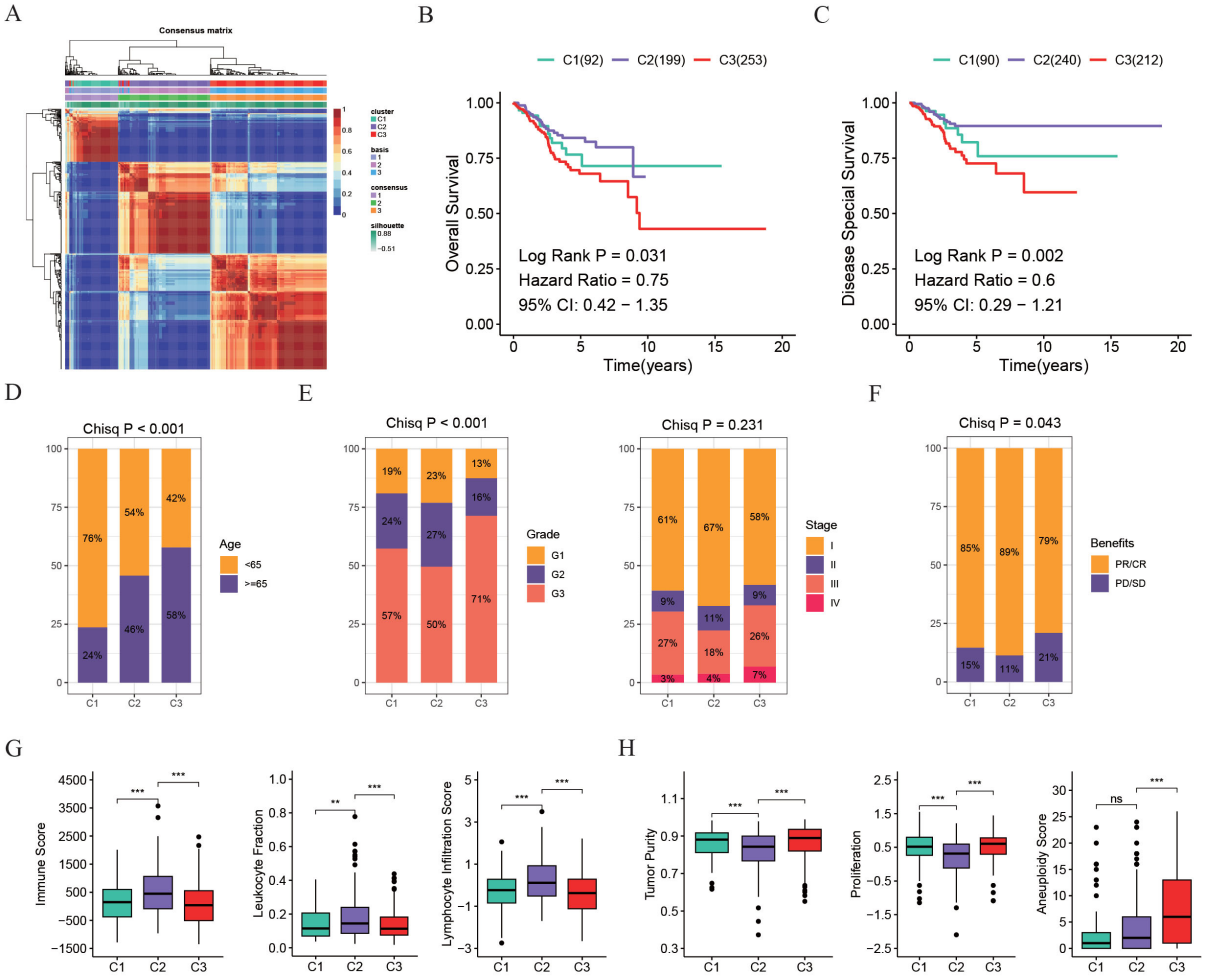
Disulfidptosis-associated genotyping of uterine corpus endometrial carcinoma. **(A)** Connectivity matrix for UCEC patients in the TCGA cohort with NMF clustering when K = 3. **(B)** Kaplan-Meier survival estimates for overall survival among different patient groups in UCEC. **(C)** Kaplan-Meier survival estimates for disease-specific survival among different patient groups in UCEC. **(D)** Age distribution across the three groups. **(E)** Distribution of tumor grade and stage across the three groups. **(F)** Distribution of chemotherapy benefits across the three groups. **(G)** Box plots showing comparisons of immune score, leukocyte fraction, and lymphocyte infiltration score among the three groups. **(H)** Box plots showing comparisons of tumor purity, proliferation, and aneuploidy score among the three groups. **p < 0.01, ***p < 0.001, ns, not significant.

Using NMF clustering analysis with the expression matrix of genes related to disulfidptosis, UCEC patients were categorized into three subgroups (**Figure 1A**). These UCEC groupings displayed noticeable disparities in OS (P=0.031) and DSS (P=0.002) (**Figure 1B**). Notably, cluster 2 exhibited a positive survival outcome, whereas cluster 3 displayed the poorest survival rates (**Figures 1B, C**). Moreover, an evaluation of the patients’ clinical features in various clusters revealed that 58% of individuals in cluster 3 were above 65 years old, contrasting with 76% in cluster 1 who were younger than 65 years (**Figure 1D**). Additionally, a higher percentage of patients in cluster 3 were diagnosed with G3 or Stage III/IV, signifying an advanced disease severity associated with a quicker decline in survival (**Figure 1E**). Furthermore, an analysis of chemotherapy response rates illustrated that patients in cluster 3 were less responsive to treatment, exhibiting higher PD/SD rates compared to cluster 1 and 2 (**Figure 1F**). Examination of immunoinfiltration and malignancy markers indicated that cluster 2 had elevated immune scores, leukocyte fractions, and lymphocyte infiltration levels, while cluster 3 had increased tumor purity, proliferation rates, and aneuploidy scores (**Figures 1G, H**).

To summarize, cluster 2 individuals exhibit a greater presence of immune-infiltrating cells, whereas cluster 3 individuals display heightened levels of malignancy and increased proliferation capabilities.

### Tumor microenvironment and immunotherapy response in three clusters

**Figure 2:**
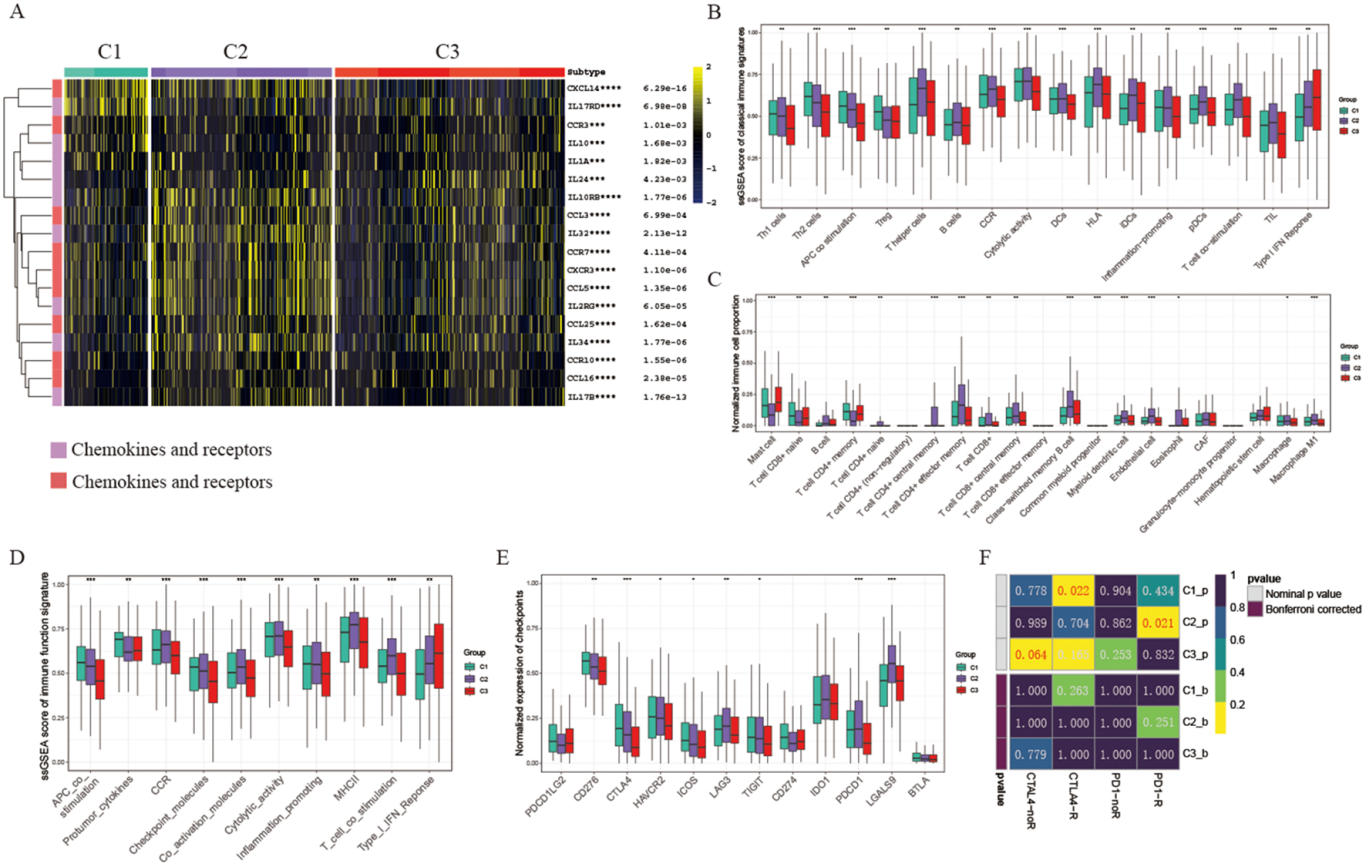
Tumor microenvironment and immunotherapy response in different clusters. **(A)** Heatmap displaying chemokine and interleukin expression across three groups. **(B)** Box plots comparing ssGSEA scores of classical immune signatures among the three groups. **(C)** Box plots comparing normalized immune cell proportions among the three groups. **(D)** Box plots comparing ssGSEA scores of immune function signatures among the three groups. **(E)** Box plots comparing normalized expression of checkpoint signatures among the three groups. **(F)** Predicted response rates of different clusters to immune checkpoint inhibitors (CTLA4 and PD1, R: Response, noR: no Response). *p < 0.05, **p < 0.01, ***p < 0.001, ****p < 0.001. blank: not significant.

Immunotherapy is the leading therapeutic approach for advanced cancers with distinct patient clusters showing unique immunoinfiltration scores. We investigated the tumor microenvironment in 3 different clusters. Tertiary lymphoid structures (TLS) act as hubs for immune cells within the TME, leading us to examine the expression profiles of various chemokines crucial for TLS development. Our analysis revealed that most of these chemokines were highly expressed in clusters 1 and 2. Specifically, chemokines such as CCR7, CCL3, CXCR3, and CCL5 were predominantly expressed in cluster 2, whereas CXCL14, and CCR3 showed the highest levels in cluster 1 (**Figure 2A**). Moreover, acknowledging that most interleukins and their corresponding receptors are associated with immune-stimulating transcripts, our research delved into the examination of the levels of these components across the groupings. The findings revealed that interleukins and their receptors showed a greater prevalence in clusters 1 and 2 when contrasted with cluster 3, which correlates with the elevated presence of chemokines and increased infiltration of immune cells in the tumor microenvironment of these clusters (**Figure 2A**). To summarize, the levels of chemokines related to tumor-infiltrating lymphocytes were notably higher in clusters 1 and 2 compared to cluster 3, indicating their role in modulating immune cell infiltration in the tumor microenvironment.

Consistently, we observed that cytolytic activity, Human Leukocyte Antigen (HLA), T cell co-stimulation, and various classical immune associated markers were predominant in clusters 1 and 2 (**Figure 2B**). The percentage of key immune cells, including B cells, CD4+ T cells, and CD8+ T cells, was notably higher in cluster 1 and 2 (**Figure 2C**). Moreover, an analysis of immune function scores in each sample revealed reduced levels of immune-related functions (e.g., Inflammation promoting, MHCII, Type I IFN Response) in cluster 3, indicating a suppression of these functions (**Figure 2D**). Considering the crucial role of immune checkpoint expression as a foundation for therapy with immune checkpoint inhibitors (ICIs), we conducted an in-depth analysis of immune checkpoint expression across five clusters. Particularly, most immune checkpoints (such as PDCD1/PD-1, CTLA4, HAVCR2/TIM-3, LAG3) showed increased expression levels in clusters 1 and 2 compared to cluster 3. Specifically, CTLA4 was predominantly expressed in cluster 1, while PDCD1 was most highly expressed in clusters 2 and 4, indicating that these clusters might be more responsive to immunotherapy (refer to **Figure 2E**). Based on these observations, we hypothesize that cluster 1 and cluster 2 could respond differently to various immunotherapy strategies. Therefore, we proceeded to evaluate the ICI response across different clusters. Our findings revealed that cluster 1 demonstrated a superior response to CTLA4 antibodies, whereas cluster 2 exhibited an enhanced response to PD-1 antibodies, in contrast to cluster 3, which showed relative resistance (**Figure 2F**).

Taken together, our results indicated that the 3 clusters mentioned above exhibit distinct characteristics within the tumor microenvironment. Overall, cluster 1 and 2 TMEs show support for anti-tumor immune responses and increased expression levels of immune checkpoint proteins, making them more conducive to benefiting from immune checkpoint inhibitors.

### Genomics variation pattern and function enrichment of different clusters

**Figure 3:**
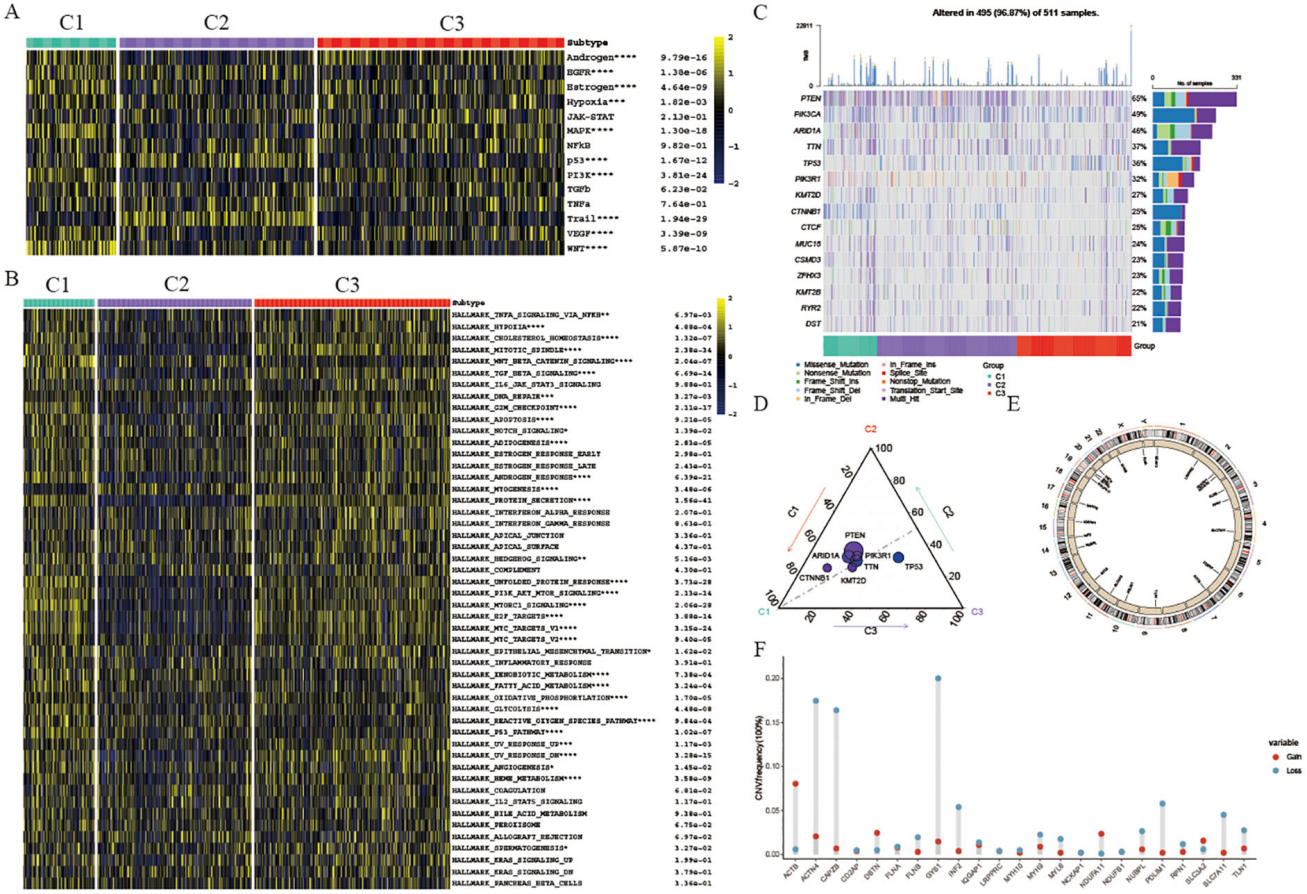
Genomics variation pattern and function enrichment of different clusters. **(A)** Heatmap showing pathway activity calculated by PROGENy among the three groups. **(B)** Heatmap displaying GSVA scores of HALLMARK pathways among the three groups. **(C)** Waterfall chart depicting the somatic mutation frequency and specific mutations of the top 15 mutated genes in the three UCEC clusters. **(D)** Mutation count of the 15 genes in each subtype. Circle size represents the number of mutations; greater distance from the subtype indicates fewer mutations. **(E)** Circle plot illustrating copy number variation of disulfidptosis-related genes. **(F)** Lollipop chart showing the gain or loss of disulfidptosis-related genes in UCEC. *p < 0.05, **p < 0.01, ***p < 0.001, ****p < 0.001, blank: not significant.

To comprehend the underlying causes for the unique prognosis and immune environments among these clusters, we conducted an analysis of cancer-related pathway activity utilizing the PROGENy R package. Our findings revealed that the enriched pathways exhibited significant variation across all three clusters. Specifically, clusters 1 and 3 displayed heightened activity in EGFR, MAPK, and WNT pathways, aligning with their poorer prognosis (**Figure 3A**). Furthermore, an examination of hallmark-related pathways through GSEA analysis revealed consistent disparities in enriched pathways among all three clusters. Notably, clusters 1 and 3 exhibited greater enrichment in oncogenic signaling pathways or targets, such as TNFA signaling, PI3K/AKT/MTOR signaling, and TGFβ signaling; pro-tumor signaling pathways, such as Epithelial-mesenchymal transition signaling; proliferation pathways, including G2M signaling, E2F signaling, MTORC1 signaling, and MYC signaling, offering a plausible rationale for clusters 1 and 3 displaying a lower survival rate and a pro-tumor microenvironment (**Figure 3B**). Additionally, considering that disulfidptosis is induced by a substantial consumption of intracellular NADPH and an abnormal accumulation of cystine and other disulfide compounds, we further investigate the activities of metabolically related pathways across the three clusters. Our findings reveal that the activity of the pentose phosphate pathway (PPP) in clusters 1 and 3 is significantly higher than that in cluster 2 (**Supplementary Figure S1A**). This suggests that tumor cells in patients belonging to cluster 2 are more susceptible to disulfide death, which further elucidates why cluster 2 patients exhibit a more effective self-clearance mechanism for tumor cells, resulting in a higher survival rate.

Somatic alterations such as mutations and variations in gene copy numbers (CNV) as key players in both promoting antitumor immune response and shaping tumor progression, our study focused on the top 15 genes with the most frequent mutations within these three distinct clusters. Notably, PTEN displayed the highest mutation frequency across all three clusters (**Figure 3C**). Specifically, cluster 1 exhibited a notably elevated frequency of CTNNB1 mutations compared to cluster 2 and cluster 3 clusters, with cluster 1 also displaying a significantly higher TP53 mutation frequency (**Figure 3D**). TP53, a well-known tumor suppressor gene, when mutated, is associated with increased tumor cell proliferation and invasion, aligning with the poorer survival outcomes observed in cluster 3 patients. Conversely, cluster 2 did not exhibit a significantly higher mutation rate in these genes, potentially explaining the better survival outcomes seen in this group. Furthermore, our analysis of CNV in genes related to disulfidptosis within the three clusters revealed noteworthy findings (**Figure 3E**). Specifically, we observed significant copy number deletions in ACTN4, CAPZB, and GYS1 among UCEC patients, while ACTB demonstrated copy number amplification (**Figure 3F**).

### Identification of diverse neoplastic modules and dissection of tumor microenvironment based on single cell transcriptome

**Figure 4:**
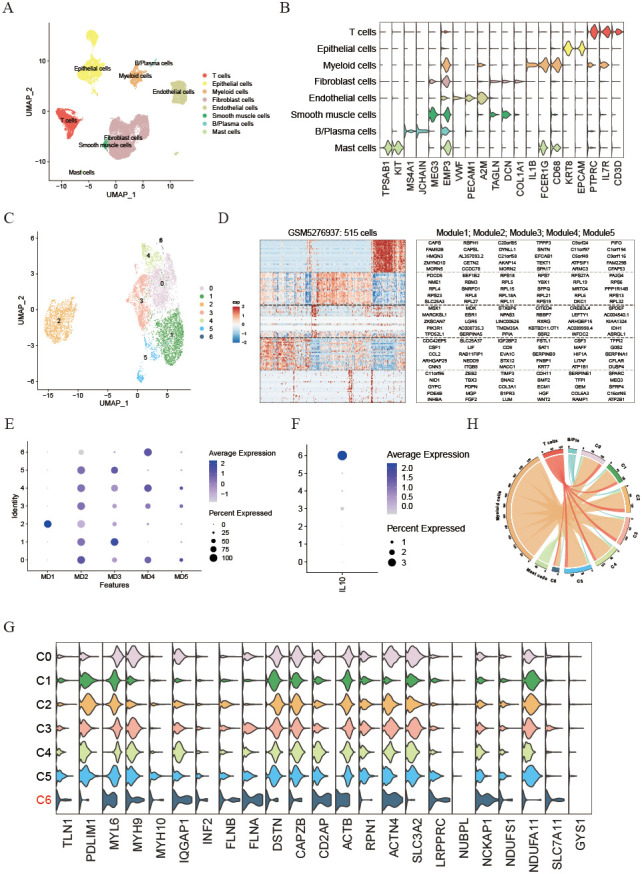
Identification of diverse neoplastic modules and dissection of tumor microenvironment based on single cell transcriptome. **(A)** UMAP plot illustrating the main cell types derived from UCEC tissues. **(B)** Violin plot displaying the expression of cell markers across each cell type. **(C)** UMAP plot showing the seven primary malignant cell types from UCEC tissues. **(D)** Heatmap of expression programs in a representative patient using NMF. **(E)** Dot plot indicating relative scores of meta-programs in each UCEC malignant cell cluster. **(F)** Dot plot showing the expression of IL10 in each UCEC malignant cell cluster. **(G)** Violin plot illustrating the expression of disulfidptosis-related genes in each malignant cell cluster. **(H)** Circle plot depicting the number of cell communication pairs between malignant and immune cell clusters.

To investigate how disulfidptosis affects the tumor microenvironment, single-cell mRNA profiles from five UCEC tissues in the GSE173682 dataset were analyzed. After implementing quality control procedures, cells were categorized into eight unique cell types utilizing conventional biomarkers and the singleR R package (see **Figure 4A**). The identified cell types include T & NK cells (CD3D, IL7R, and PTPRC), Epithelial cells (EPCAM and KRT8), Myeloid cells (CD68, FCER1G, and IL1B), Fibroblasts (COL1A1, DCN, and TAGLN), Endothelial cells (A2M, PECAM1, and VWF), Smooth muscle cells (MEG3 and EMP3), B/Plasma cells (JCHAIN and MS4A1), and Mast cells (KIT and TPSAB1) (**Figure 4B**). We then utilized the inferCNV R package to distinguish neoplastic from epithelial cells based on the reference cell types (**Supplementary Figure S2A**). Furthermore, to explore the impact of disulfidptosis on malignant UCEC cells, malignant cells were classified and divided into 7 distinct clusters (**Figure 4C**). Additionally, NMF was applied to define expression programs specific to each tumor by identifying co-expressed genes. These gene modules indicated by these expression programs were found to be highly activated in specific subsets of tumor cells, as demonstrated by the NMF results for a particular tumor sample, GSM5276937 (**Figure 4D**). Altogether, 32 distinct intra-tumor expression programs were discovered in the UCEC tissues, leading to the categorization of five modules (MDs) that were common across multiple tumors based on enriched pathways (**Supplementary Table S1**). MD1 was distinguished by an enhancement of the myogenesis pathway. MP2 included pathways related to proliferation such as myc target v1 and mtorc1 signaling. Moreover, MD3 was associated with estrogen response late, MD4 with inflammatory responses, and MD5 with epithelial mesenchymal transition characteristics (**Supplementary Figure S2B**). Interestingly, it was observed that neoplastic cells in cluster C6 exhibited enrichment for MD4 and high levels of IL10 expression (**Figures 4E, F**). Additionally, cluster C6 showed high levels of SLC7A11 expression, an important transporter linked to cell disulfide death, implicating that malignant UCEC cells displaying increased SLC7A11 expression had heightened potential for inflammation (**Figure 4G**). Furthermore, analyses of cell-to-cell interactions utilizing the cellphoneDB algorithm indicated that the number of intercellular interactions between highly disulfidptosis malignant UCEC cells and immune-associated cells were notably reduced compared to other malignant cells (**Figure 4H**). This insight suggests that malignant cells with elevated SLC7A11 expression could indirectly stimulate inflammatory responses and stimulate the expression of inflammatory factor IL10 while decreasing interactions with immune cells.

### Establishment of a disulfidptosis model for predicting the prognosis and immunotherapy response of UCEC patients

**Figure 5:**
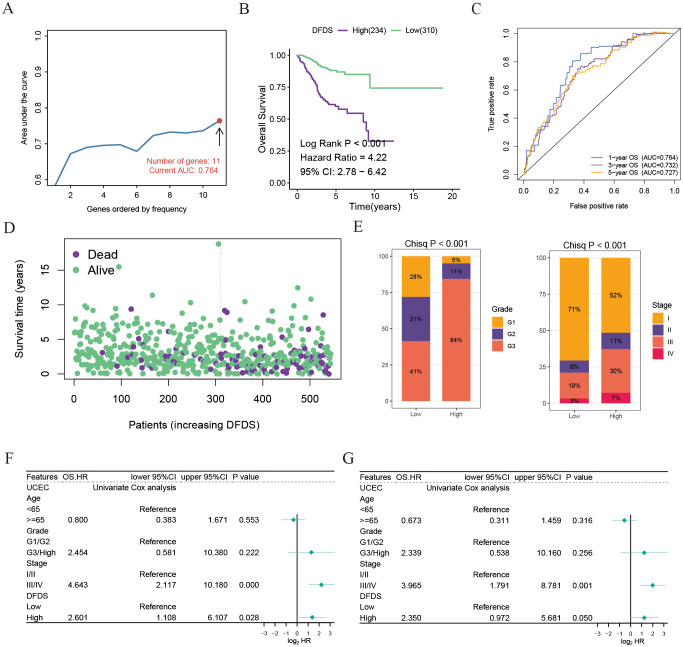
Establishment of a disulfidptosis model for predicting the prognosis and immunotherapy response of UCEC patients. **(A)** AUC values of Lasso and Cox models using different numbers of disulfidptosis-related genes. **(B)** Kaplan-Meier survival estimates for patients with high and low DFDS. **(C)** ROC curves for DFDS predicting 1-year, 3-year, and 5-year prognosis. **(D)** Distribution of DFDS and survival status in UCEC patients. **(E)** Proportions of different grades and stages in high and low DFDS groups. **(F, G)** Univariate and multivariate Cox analyses evaluating the independent prognostic value of DFDS in UCEC patients.

The above results have validated a connection between disulfidptosis and the outlook, benefits of immunotherapy, and response to chemotherapy in UCEC patients. This suggests that genes related to disulfidptosis could be utilized in the assessment of prognosis and response to treatment. To achieve this, we conducted a screening of 23 genes related to disulfidptosis to establish a model for the assessment of disulfidptosis score (DFDS) using a LASSO regression model (**Figure 5A**). Subsequently, we computed the DFDS for each patient and categorized them into high- and low- DFDS groups based on an optimal threshold determined using the ‘survminer’ R package. Evidently, Kaplan-Meier analysis indicated that patients with high-DFDS exhibited a lower survival rate in comparison to those with low-DFDS, which was also validated in GSE9891 (**Figure 5B** and **Supplementary Figure S3A**). Furthermore, the ROC curve illustrated that the DFDS model displayed commendable sensitivity and specificity in predicting the risk of survival [AUC = 0.764 (1 year), 0.732 (3 years), 0.727 (5 years) (**Figure 5C**)], a finding supported by the mortality rates in both groups (**Figure 5D**). Additionally, a correlation was identified between DFDS and the clinical characteristics of UCEC patients, specifically demonstrating that higher DFDS levels were linked with more advanced stages of the disease (**Figure 5E**). Subsequent analysis using a Cox regression model to evaluate the performance of the DFDS model in conjunction with other clinical features (such as age, pathological type, and pathological stage) revealed that our DFDS model exhibited superior performance in comparison to age, grade, and stage, functioning as an independent prognostic factor for predicting the outcomes of UCEC patients (**Figures 5F, G**).

Collectively, our findings indicate that this DFDS model shows great promise as a reliable biomarker for evaluating clinical results in patients with UCEC.

### High DFDS suppressed the immune function pathways and insensitive to immunotherapy and chemotherapy

**Figure 6:**
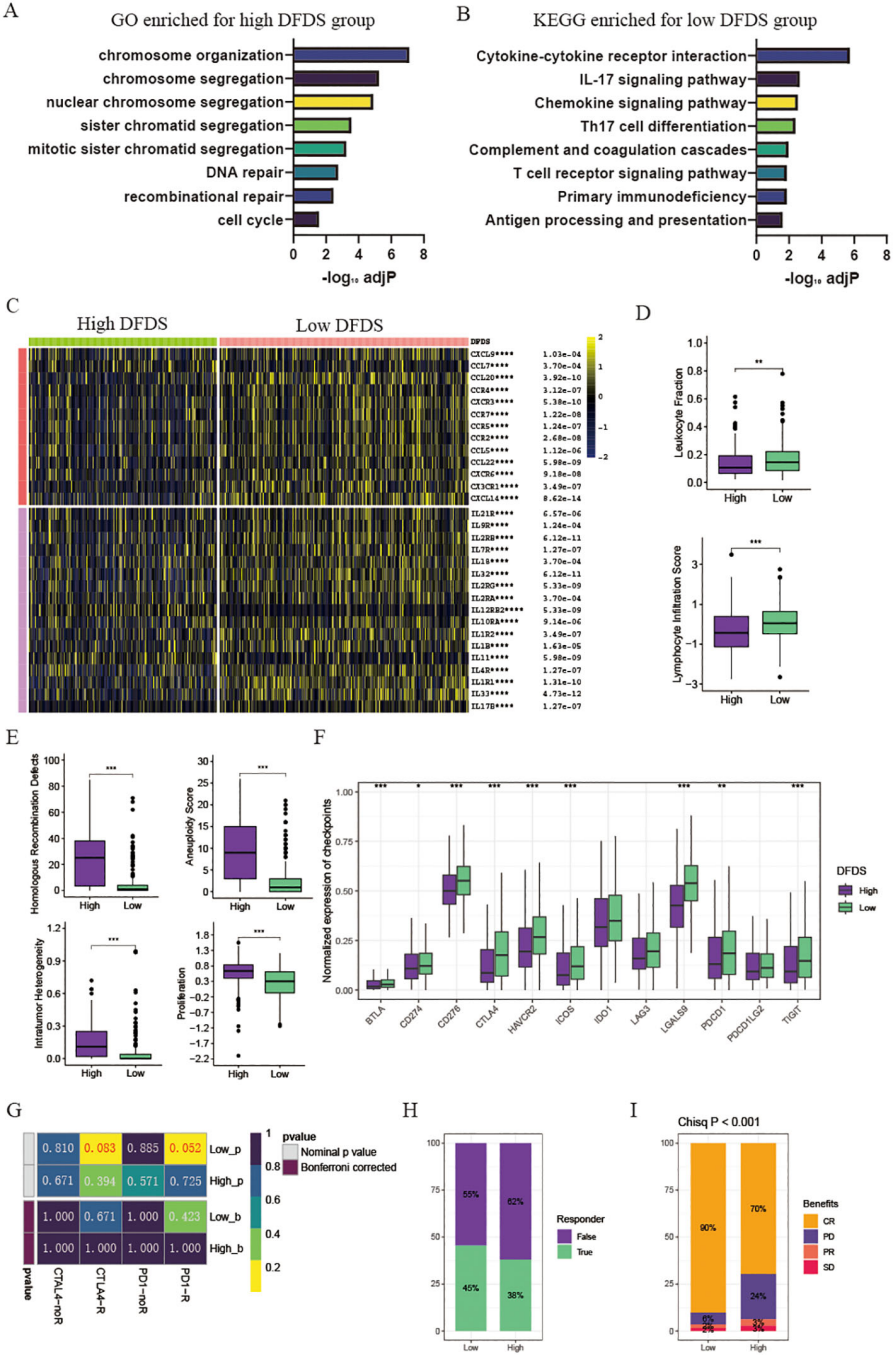
Tumor microenvironment and pathway enrichment in different DFDS groups. **(A)** Barplot illustrating GO enriched pathways for the high DFDS group. **(B)** Barplot displaying KEGG enriched pathways for the low DFDS group. **(C)** Heatmap showing the expression levels of chemokines and interleukins in high and low DFDS groups. **(D)** Box plots comparing leukocyte fraction and lymphocyte infiltration scores between high and low DFDS groups. **(E)** Box plots comparing homologous recombination defects, aneuploidy scores, intratumor heterogeneity, and proliferation scores between high and low DFDS groups. **(F)** Box plots comparing the expression of immune checkpoints between high and low DFDS groups. **(G)** Predicted response rates of different DFDS groups to immune checkpoint inhibitors. **(H)** Proportions of immunotherapy response in high and low DFDS groups. **(I)** Proportions of chemotherapy benefits in high and low DFDS groups. Response includes CR/PR, and Non-response includes PD/SD (CR, complete response; PD, progressive disease; PR, partial response; SD, stable disease). *p < 0.05, **p < 0.01, ***p < 0.001, ****p < 0.001, blank: not significant.

To investigate the microenvironment of high-DFDS and low-DFDS groups and to assess its accuracy in predicting immunotherapy response, we first performed a GSEA based on the differentially expressed genes between these cohorts. In the high-DFDS group, pathways associated with treatment resistance and tumor proliferation, including DNA repair and cell cycle pathways, were notably upregulated (**Figure 6A**). In contrast, pathways pertaining to immune functions, such as Cytokine-cytokine receptor interaction, T cell receptor signaling, Primary immunodeficiency, and Antigen processing and presentation, were significantly upregulated in the low-DFDS group, which corresponds with better survival outcomes for low-DFDS patients (**Figure 6B**). Additionally, we evaluated the expression levels of chemokines, their receptors, interleukins, and their respective receptors, as well as immune cell infiltration levels, malignancy scores, and immune checkpoint expressions in both high- and low-DFDS groups to determine the model’s efficacy in predicting immunotherapy responses. We found that most chemokines, interleukins, and their receptors were considerably higher in the low-DFDS group compared to the high-DFDS group (**Figure 6C**). Moreover, leukocyte and lymphocyte infiltration scores were elevated in the low-DFDS group, suggesting that immune function was suppressed in the high-DFDS group (**Figure 6D**). Furthermore, elevated levels of homologous recombination defects, intratumoral heterogeneity, aneuploidy scores, and proliferation scores were observed in the high-DFDS group, which correlates with poorer survival outcomes for patients in this cohort (**Figure 6E**). Importantly, the expression of most immune checkpoints, such as PDCD1, CTLA4, and HAVCR2, was higher in the low-DFDS group, indicating that patients in the low-risk group may benefit more from immunotherapy (**Figure 6F**).

The results above demonstrated that the potential prediction of immunotherapy effects by the DFDS model. Following this, assessing the sensitivity of high-DFDS and low-DFDS patients to PD-1 and CTLA4 immunotherapy was conducted. The findings showed that individuals in the low-DFDS group had a higher response rate to PD-1 immunotherapy, with a greater number of responders compared to those in the high-DFDS group (**Figures 6G, H**). Additionally, the response to chemotherapy in advanced cancer patients treated with a combination of chemotherapy and immunotherapy was analyzed between the high-DFDS and low-DFDS categories. It was observed that patients in the low-DFDS group exhibited an enhanced sensitivity to chemotherapy (**Figure 6I**).

Overall, our findings indicated that this DFDS model serves as a promising and robust biomarker for evaluating clinical outcomes and responses to immunotherapy responses in UCEC patients with UCEC.

### LRPPRC server as a novel biomarker and promotes the proliferation and invasion abilities of UCEC

**Figure 7:**
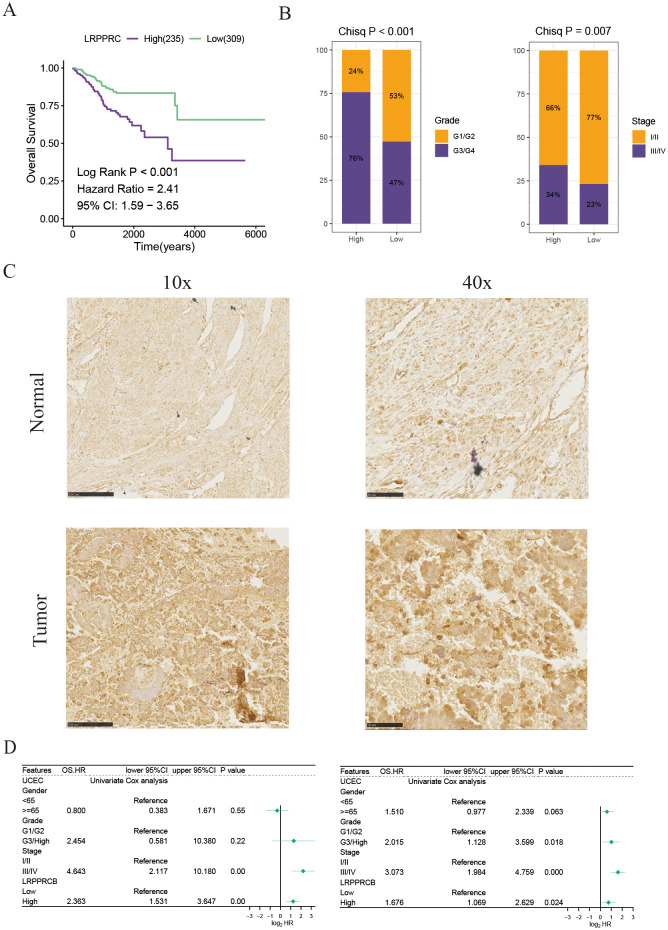
LRPPRC expression is associated with the prognosis of patients with UCEC. **(A)** Kaplan-Meier survival estimates for patients with high and low LRPPRC expression. **(B)** Proportions of different grades and stages in high and low LRPPRC groups. **(C)** Representative IHC staining of LRPPRC in paracancerous and tumor UCEC samples. **(D)** Univariate and multivariate Cox analyses evaluating the independent prognostic value of LRPPRC in UCEC patients.

To strengthen the validity of the DFDS model as a practical clinical tool, our research expanded to investigate the genes linked to disulfidptosis in this model. Using univariate Cox regression analysis, we determined that only five of these genes exhibited significant associations with UCEC prognosis (**Figure 7A** and **Supplementary Figure S3B**). Of note, the gene LRPPRC, known as Leucine Rich Pentatricopeptide Repeat Containing, was specifically linked to the advancement of tumor grade and stage (**Figure 7B** and **Supplementary Figures S3C, D**). Previous study has established that LRPPRC is often upregulated in urothelial carcinoma of the bladder, where it regulates redox balance via the circANKHD1/FOXM1 pathway to drive tumorigenesis (18). Similarly, research has suggested that LRPPRC could boost the spread and growth of malignant cells, with its role in UCEC remaining largely unexplored. We found that the expression of LRPPRC was significantly higher in UCEC malignant tumor cells compared to other cells (**Supplementary Figure S4A**). Moreover, LRPPRC exhibited the highest expression in cluster 5 tumor cells, which displayed pronounced epithelial-mesenchymal transition (EMT) characteristics (**Figure 4D** and **Supplementary Figure S4B**), suggesting that LRPPRC may play a role in promoting tumor migration and invasion. Notably, our results confirmed the heightened LRPPRC expression in UCEC tissue compared to adjacent normal tissue, as shown by immunohistochemical staining (**Figure 7C**). In addition, univariate and multivariate Cox regression analyses indicated that LRPPRC expression serves as an independent prognostic indicator (**Figure 7D**). Furthermore, a univariate Cox regression analysis of the pan-cancer cohorts revealed that LRPPRC expression was negatively correlated with prognosis across multiple cancer types, including adrenocortical carcinoma (ACC), kidney renal clear cell carcinoma (KIRC), and liver hepatocellular carcinoma (LIHC) (**Supplementary Figure S4C**). These findings enhance the reliability and consistency of our DFDS model in studies of UCEC.

**Figure 8:**
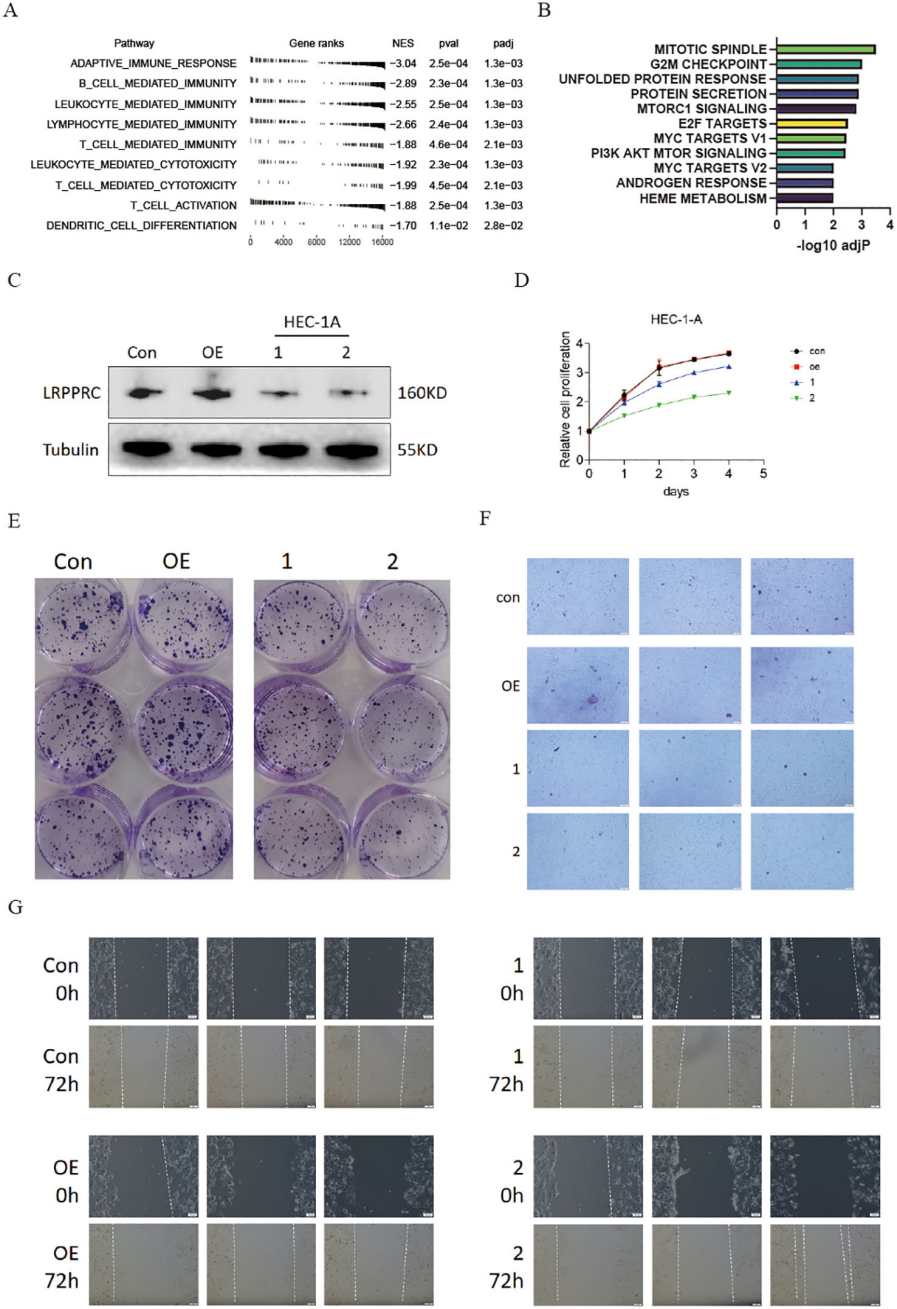
Knockdown of LRPPRC restrained the proliferation and invasion abilities of UCEC. **(A)** GO analysis displaying the normalized enrichment scores (NES) of adaptive immune response and immune cell-related pathways in UCEC patients with high LRPPRC expression. Negative NES indicates a negative correlation between the pathway and LRPPRC expression. **(B)** GSEA analysis showing enrichment of HALLMARK signaling pathways or processes in UCEC patients with high LRPPRC expression. **(C)** Western blot validation of LRPPRC knockdown in HEC-1A cells. **(D)** Proliferation of LRPPRC knockdown versus control cells. **(E–G)** Clone formation, migration, and invasion abilities of indicated UCEC cells.

To ascertain the role of LRPPRC in the tumor microenvironment and progression of uterine corpus endometrial carcinoma, we initially examined LRPPRC’s involvement in the TME of UCEC patients by leveraging GO pathways. The analysis revealed that LRPPRC expression inversely correlated with adaptive immune responses and immune cell-mediated immunity, specifically involving B cells, leukocytes, lymphocytes, and T cells, as well as immune cell-mediated cytotoxicity (including T cells and leukocytes), T cell activation, and dendritic cell (DC) differentiation (**Figure 8A**). Furthermore, LRPPRC expression was found to heighten the activity of oncogenic and proliferation signaling pathways, indicating its immunosuppressive function in the TME of UCEC (**Figure 8B**).

Following this, we reduced the expression of LRPPRC in HEC-1A cells (**Figure 8C**). As anticipated, the reduced expression of LRPPRC markedly inhibited proliferation (**Figure 8D**), as well as the migration and invasion capacities (**Figures 8E–G**), aligning with the previously mentioned results.

## Discussion

Immunotherapy with immune checkpoint inhibitors has emerged as the standard second-line treatment for advanced cancers, with approval for first-line indications on the rise. Disulfidptosis, an abnormal accumulation of disulfide compounds leading to programmed cell death, is triggered by limited glucose availability and insufficient redox capacity (10). Past research indicates a strong connection between disulfidptosis, the tumor microenvironment, and the immune response in advanced cancers (19, 20). Nevertheless, there is currently no direct evidence establishing the relationship between disulfidptosis and the response to immune checkpoint inhibitors in UCEC.

Hence, a comprehensive analysis of multi-omic factors will be conducted to study the differences in functionality among various subclusters of disulfidptosis and its impact on the immune landscape and potential immunotherapeutic benefits. This research will greatly enhance our understanding of disulfidptosis in the field of onco-immunology by identifying populations with the highest potential for efficacy and developing effective therapeutic strategies. In this study, UCEC patients were categorized into three clusters based on genes related to disulfidptosis. Significant variations in survival rates were observed among these clusters, with cluster 2 displaying the most favorable survival results, while cluster 3 exhibited the poorest outcomes. The immune cells found in the tumor microenvironment play a crucial role in tumor growth, with distinct subgroups of immune cells linked to different kinds of tumors. Moreover, even within the same pathological type, the immune cell subpopulations may vary among patients (21, 22). Our study findings indicated that cluster 2 displayed heightened levels of immune cell penetration, including B cells, CD4T cells, CD8T cells, Tfh cells, and DCs, combined with enhanced immune functions such as cytolytic activity, T cell co-stimulation, and inflammation stimulation. Conversely, cluster 3 displayed lower levels of immune cell infiltration and suppressed immune function. CD8+T cells are known to be crucial for initiating anti-tumor immune responses (23). TILs play a crucial role in tumor rejection by identifying tumor antigens and eliminating transformed cells. Meanwhile, Tfh cells contribute to the development of tertiary lymphoid structures near tumors, thereby enhancing anti-tumor immunity (24). On the other hand, Treg cells facilitate the evasion of tumor cells from immune surveillance by inhibiting the cytotoxic activity of killer T cells, ultimately facilitating tumor progression (25, 26). These variations in immune infiltration correspond to disparities in survival rates. The use of immune checkpoint inhibitors is a promising strategy for managing advanced cancer patients (27). Elevated expression levels of immune checkpoints are associated with improved responses to such inhibitors. Our analysis revealed an upregulation of key immune checkpoints in clusters 1 and 2, while a downregulation in cluster 3. Notably, patients in clusters 1 and 2 exhibited enhanced responses to ICIs, with cluster 1 demonstrating a poorer survival rate compared to cluster 2, likely due to the tumor microenvironment favoring pro-tumor immunity involving TNFA, PI3K/AKT/MTOR, and TGFβ signaling pathways (28, 29). Furthermore, we observed distinct somatic variations among the different clusters, with cluster 3 demonstrating a higher frequency of TP53 mutations. Clinical evidence indicates that advanced cancer patients with TP53 mutations have a worse prognosis and are more resistant to chemotherapy (30, 31). Additionally, through single-cell transcriptome analysis, we investigated the impact of disulfidptosis on malignant cells, revealing that disulfidptosis-positive tumor cells display increased inflammation potential and reduced communication with immune cells.

Furthermore, a disulfide scoring model was developed to forecast the prognosis of UCEC. The results of the model exhibited a commendable AUC among UCEC patients. In the analysis by Kaplan-Meier, those with elevated DFDS exhibited notably diminished survival rates and higher tumor grades in comparison to those with lower DFDS. Additionally, the constructed DFDS was identified as an independent prognostic factor. Our model displayed superior predictive capabilities for survival in contrast to conventional clinical and pathological factors. In conclusion, LRPPRC, a scarcely studied protein in cancer research, was found to have a connection to worse survival rates among UCEC patients. It is worth mentioning that increasing LRPPRC levels notably boosted the growth and invasion abilities of UCEC cells, indicating its potential as a valuable target for treating UCEC.

To conclude, our study delineated the landscape of disulfidptosis-related transcriptomics, genetic changes, and immune infiltration. Notably, we developed a novel classification system along with a precision model designed to assess both the prognosis and advantages of immunotherapy. This serves as a valuable resource to improve decision-making processes and monitoring protocols for individual UCEC patients. Moreover, we identified a new biomarker, LRPPRC, which can predict the prognosis of UCEC patients. Although we have shown the exceptional predictive accuracy of disulfidptosis in evaluating UCEC prognosis and immunotherapy benefits, it is crucial to recognize certain limitations in our research. The prognostic and immunotherapeutic predictions of the DFDS model and classification are based on algorithmic projections, and further validation using actual UCEC immunotherapy cohorts is necessary.

## Data Availability

The original contributions presented in the study are included in the article/**Supplementary Material**. Further inquiries can be directed to the corresponding authors.

## Supplementary material

Supplementary Figure 1 Pathway enrichment of different subgroups. **(A)** Heatmap showing the GSVA score of metabolic pathways.

Supplementary Figure 2 Identification of malignant cells from UCEC epithelial cells. **(A)** Heatmap showing copy number variation of reference cells and malignant epithelial cells. **(B)** Heatmap depicting shared expression meta-programs across all patients.

Supplementary Figure 3 Validation of DFDS model and LRPPRC negative correlated with the survival of UCEC. **(A)** Kaplan-Meier survival estimates for patients with high and low DFDS in GSE9891. **(B)** Univariate and analyze evaluating the prognostic value of genes used in DFDS. **(C)** Box plots comparing expression of 5 genes among different grades. **(D)** Box plots comparing expression of 5 genes among different stages.

Supplementary Figure 4 Single cell and pan-cancer cohorts validated the function of LRPPRC. **(A)** Dot plot showing the expression of LRPPRC in each UCEC cell types. **(B)** Dot plot showing the expression of LRPPRC in each UCEC malignant cell types. **(C)** Univariate and Cox analyses evaluating the prognostic value of LRPPRC in pan-cancer cohorts.
