# Supplementary figures and images for: Integrated analysis and experiments uncover the function of disulfidptosis in predicting immunotherapy effectiveness and delineating immune landscapes in uterine corpus endometrial carcinoma
Source: Front Immunol. 2024 Oct 9;15:1454730. doi: 10.3389/fimmu.2024.1454730 (PMC11496088; doi:10.3389/fimmu.2024.1454730)

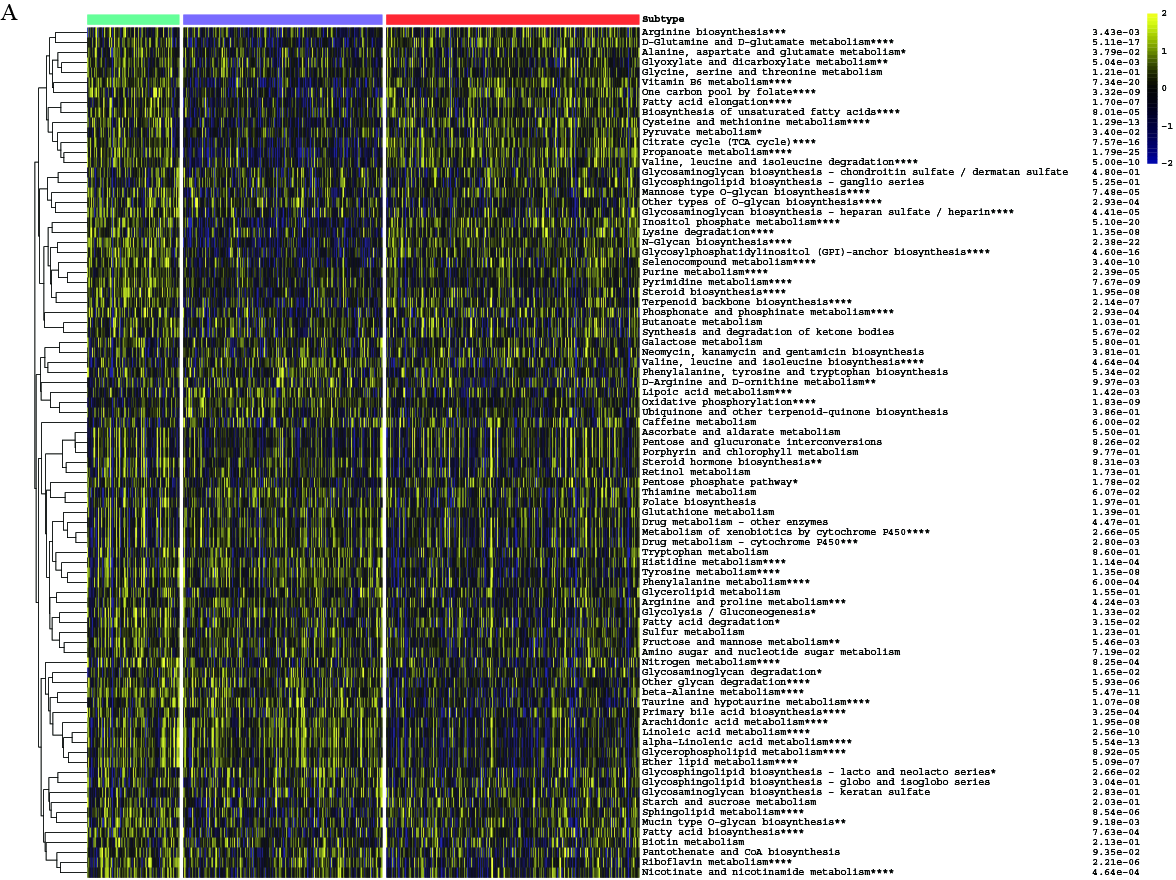

Supplement: Supplementary Figure 1 — Pathway enrichment of different subgroups. (A) Heatmap showing the GSVA score of metabolic pathways. [file Image1.tif]

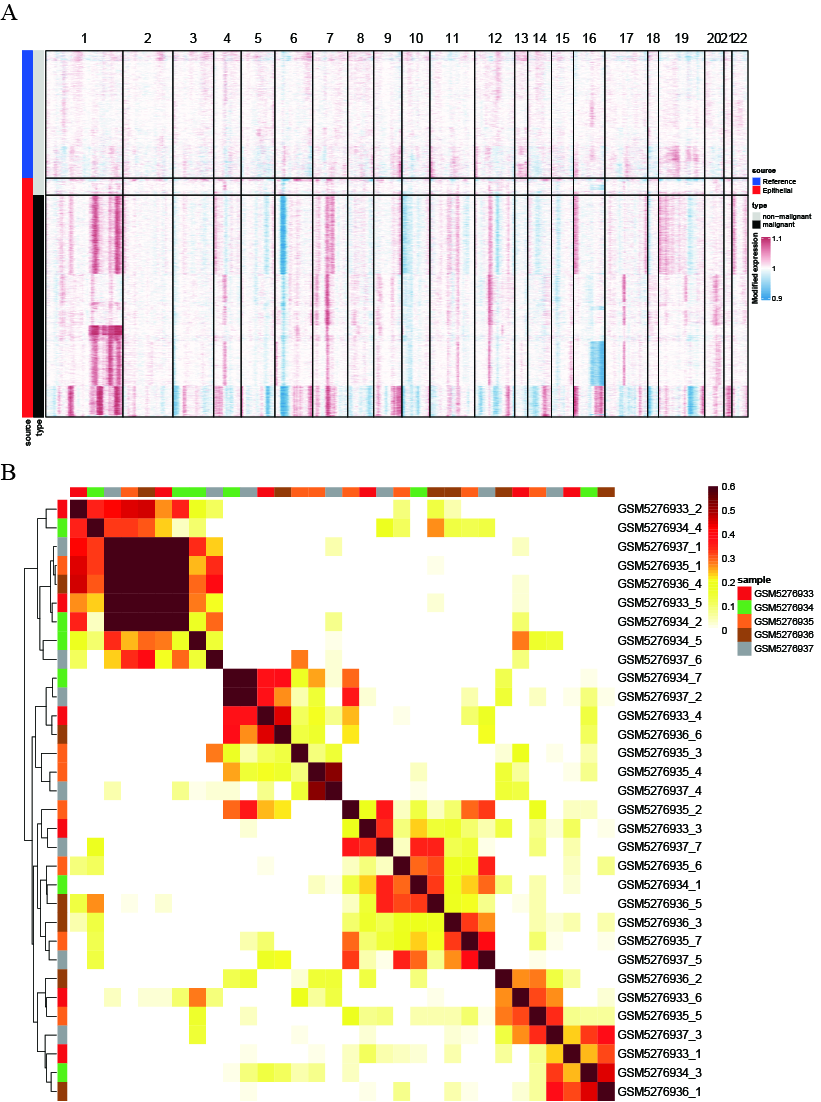

Supplement: Supplementary Figure 2 — Identification of malignant cells from UCEC epithelial cells. (A) Heatmap showing copy number variation of reference cells and malignant epithelial cells. (B) Heatmap depicting shared expression meta-programs across all patients. [file Image2.tif]

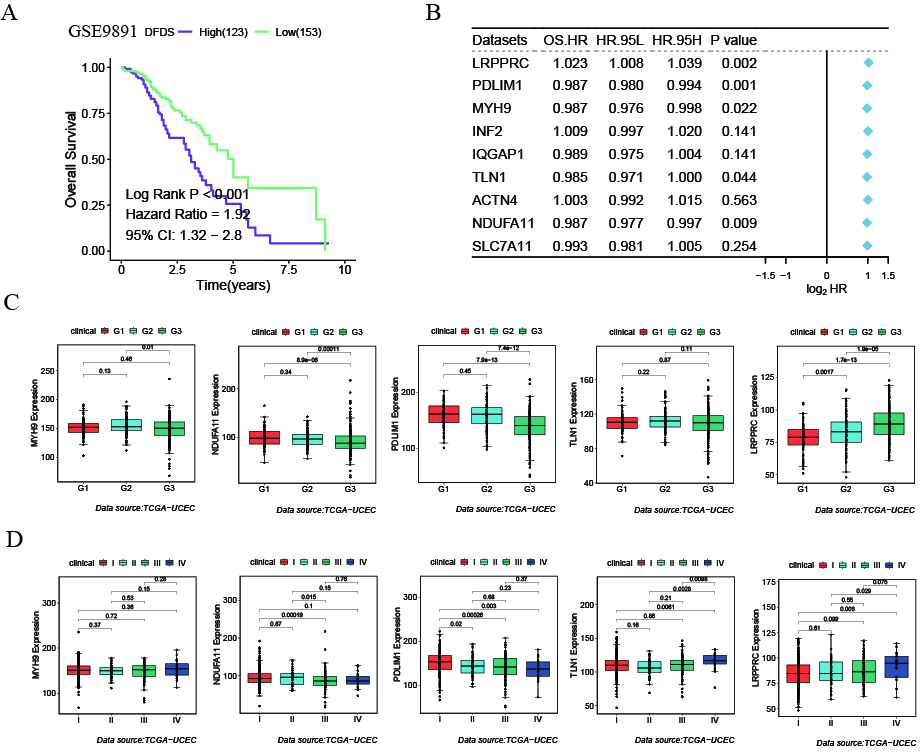

Supplement: Supplementary Figure 3 — Validation of DFDS model and LRPPRC negative correlated with the survival of UCEC. (A) Kaplan-Meier survival estimates for patients with high and low DFDS in GSE9891. (B) Univariate and analyze evaluating the prognostic value of genes used in DFDS. (C) Box plots comparing expression of 5 genes among different grades. (D) Box plots comparing expression of 5 genes among different stages. [file Image3.tif]

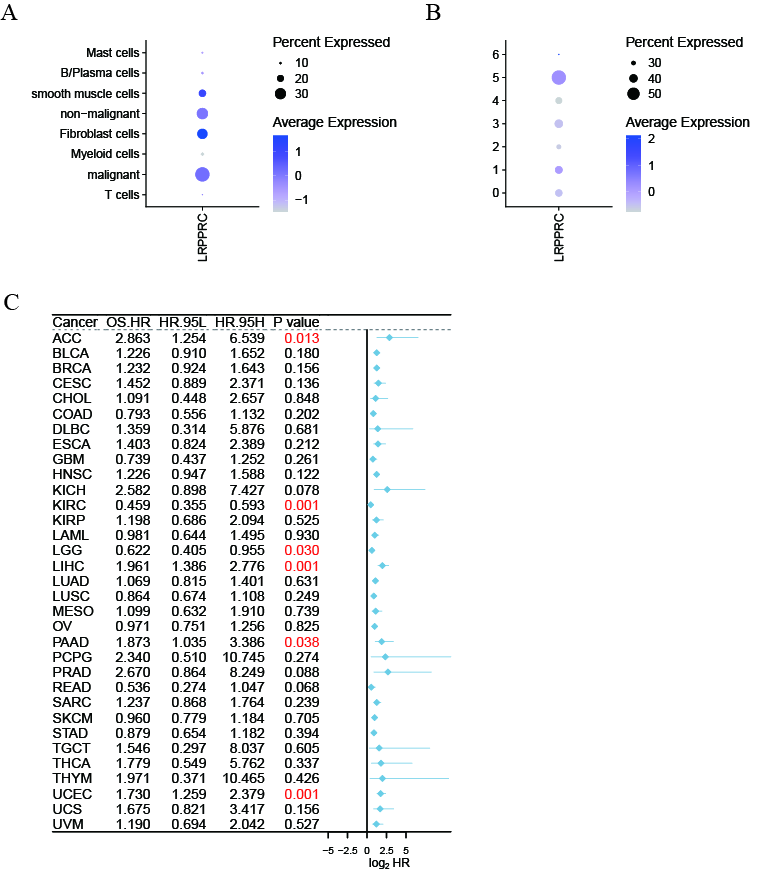

Supplement: Supplementary Figure 4 — Single cell and pan-cancer cohorts validated the function of LRPPRC. (A) Dot plot showing the expression of LRPPRC in each UCEC cell types. (B) Dot plot showing the expression of LRPPRC in each UCEC malignant cell types. (C) Univariate and Cox analyses evaluating the prognostic value of LRPPRC in pan-cancer cohorts. [file Image4.tif]
